# Unveiling Progress: A Systematic Review and Meta-Analysis of Endoscopic Posterior Cricoid Split With Posterior Cartilage Graft

**DOI:** 10.7759/cureus.73830

**Published:** 2024-11-16

**Authors:** Abdullah Sindi, Alhanouf Alhedaithy, Nasser Almutairi, Waleed Alshareef, Abdullah Aljasser, Ahmad Alammar

**Affiliations:** 1 Otolaryngology - Head and Neck Surgery, King Saud University Medical City, Riyadh, SAU; 2 Otolaryngology - Head and Neck Surgery, King Abdullah Medical Complex, Jeddah, SAU; 3 Otolaryngology - Head and Neck Surgery, King Fahad Medical Military Complex, Dhahran, SAU; 4 Otolaryngology, Alfaisal University College of Medicine, Riyadh, SAU; 5 Otolaryngology - Head and Neck Surgery, King Faisal Specialist Hospital & Research Centre, Riyadh, SAU; 6 Otolaryngology - Head and Neck Surgery, Maternity and Children's Hospital, Makkah, SAU

**Keywords:** endoscopic, laryngotracheal reconstruction, meta-analysis, posterior cartilage graft, subglottic stenosis, vocal fold immobility

## Abstract

Subglottic and posterior glottic stenosis (PGS) narrows distinct areas of the larynx, while bilateral vocal fold immobility (BVFI) is characterized by static cords. Treatments include open surgeries and newer endoscopic methods, offering comparable safety, quicker recovery, and fewer complications. This study assesses the decannulation rate of endoscopic posterior cricoid split with posterior cartilage grafting (EPCCG) in pediatric patients with posterior glottic stenosis, subglottic stenosis (SGS), and BVFI. Other outcomes include complications, symptom relief, need for additional airway procedures, and hospital stay. We retrieved relevant records published between 2003 and 2024 from PubMed, Scopus, Web of Science, and Cochrane Library. Using OpenMeta v5.26.14 software, we pooled the decannulation rates from individual studies. Other outcomes reported in fewer studies than what justifies a meta-analysis were synthesized manually. The selection process yielded 15 articles, 11 of which were eligible for analysis. The decannulation rate had an estimated proportion of approximately 83.2% (95% CI: 74.0-92.4%). Complications were present in 6/70 patients, and no mortality was reported. Additional airway procedures were needed in 14/82 patients for whom the outcome was reported. Hospital stays averaged 6.1 days in four studies reporting the outcome. Symptomatic relief was achieved in most of the patients; however, some cases reported odynophagia and concerns about voicing. EPCCG shows promise in treating less severe cases of PGS, SGS, and BVFI, offering safety, short hospital stays, and symptomatic relief. However, its efficacy for advanced cases and comorbidities needs more research. The limitations, including multiple pathologies and comorbidities in patients, hinder broader applicability. More extensive studies with standardized protocols are required in order to overcome these limitations.

## Introduction and background

Subglottic stenosis (SGS) and posterior glottic stenosis (PGS) are distinct causes of airway compromise affecting different parts of the larynx. SGS refers to a narrowing of the airway in the subglottic region, which extends from below the vocal cords to the upper trachea. On the other hand, PGS is defined as a constriction at the posterior portion of the true vocal folds within the glottis area [1,2]. Moreover, bilateral vocal fold immobility (BVFI) is a rare and challenging cause of airway compromise characterized by an inability of vocal cords to move [3].

SGS may be idiopathic in origin or can be caused by various factors, such as trauma and autoimmune diseases like granulomatosis with polyangiitis [4,5]. SGS may cause serious complications for patients, including respiratory distress, dysphonia, stridor, deglutition, and breathing. Complications may also encompass partial or complete airway occlusion and long-term sequelae, such as subglottic tracheal stenosis and bilateral vocal cord immobility [6-8]. PGS is usually caused by prolonged endotracheal intubation, leading to injury to the interarytenoid mucosa and the underlying cartilage, which can result in fibrosis, contracture, and bilateral vocal cord immobility [9-11]. PGS can even compromise the airway, leading to dyspnea and tracheostomy dependence [12]. BVFI is commonly caused by vocal fold paralysis or cricoarytenoid joint fixation, with thyroid surgery being the most common cause [13]. The condition can result in dyspnea and tracheostomy dependence and, therefore, requires careful management and treatment [9].

Many treatment modalities were developed to address these conditions. Open surgical options include procedures such as tracheotomy, total arytenoidectomy, subtotal arytenoidectomy, transverse cordectomy, vocal fold lateralization, and open and reinnervation techniques [14]. Other surgical options for PGS may involve laryngeal exposure via laryngofissure, removal of posterior glottic stenotic tissues, and then the use of autologous grafting [15]. However, open-surgical approaches have a higher risk of complications. These procedures can lead to extended recovery periods, inherent postoperative risks such as infections and bleeding, and the possible need for tracheostomy [16,17]. Additionally, scar tissue formation is risky, potentially causing recurrent stenosis and alterations in voice quality and speech [18].

Newer, less invasive techniques have seen much development in the past two decades. Endoscopic posterior cricoid split with posterior cartilage grafting (EPCCG) is a novel technique for treating SGS, PGS, and BVFI. This procedure involves splitting the posterior portion of the cricoid cartilage and placing a cartilage graft to expand the airway and improve vocal fold mobility. Rib or costal cartilage grafts are common in this approach to provide structural support and prevent restenosis [19-21].

Endoscopic methods have several advantages over open surgery, including comparable safety, shorter operative times, reduced costs, improved cosmetic results, and quicker recovery. These methods also lead to fewer wound problems, reduced blood transfusion rates, decreased postoperative complications, and improved surgical precision [22-28]. This study aims to evaluate EPCCG as the main treatment approach for PGS, SGS, and BVFI in pediatric patients. We will examine the procedure’s impact on the decannulation rate, complications, symptom relief, need for additional airway procedures, and hospital stay. By analyzing evidence from existing studies, we aim to offer comprehensive guidance for potentially incorporating this technique into the standard treatment of SGS, PGS, and BVFI.

## Review

Methods

This systematic review has followed the Preferred Reporting Items for Systematic reviews and Meta-Analyses (PRISMA) recommendations as well as the guidelines provided by the Cochrane Handbook for Systematic Reviews of Interventions [29,30].

Search Strategy

The bibliographic search was carried out in the following databases for articles published between 2003 and 2024: PubMed, Scopus, Web of Science, and Cochrane. Specific descriptors and their synonyms were used according to the Medical Subject Headings: endoscopic surgery, posterior cricoid split, cricoid cartilage, cartilage graft, airway reconstruction, laryngotracheal stenosis, airway augmentation, bilateral vocal cord paralysis, posterior glottic stenosis, and surgical techniques. The terms were associated with each other with the word "OR" resulting in the following search strategy: ("Endoscopic Posterior Costal Cartilage Grafting" OR "Endoscopic Posterior Cricoid Split" OR "Endoscopic Posterior Split" OR "Posterior Cricoid Rib Grafting" OR "Costal Cartilage Graft" OR "Posterior Cricoid Grafting" OR "EPCCG" OR "EPCS/RG").

Selection Criteria

Articles were included that focused on the endoscopic posterior cricoid split with posterior cartilage graft as a surgical intervention in children subjects diagnosed with either SGS, PGS, or bilateral vocal cord paralysis. Additionally, prospective and retrospective cohort studies, case series, and case reports were included. Studies reporting outcomes such as airway patency, decannulation rate, voice quality, swallowing function, and complications were also included. Articles primarily focused on surgical techniques other than endoscopic posterior cricoid split with posterior cartilage graft were excluded. The entire screening process was carried out by two independent reviewers after eliminating duplicate records. In case of discrepancies, a third evaluator was consulted for a final decision. After the database search, eligible articles were selected by title, abstract, and full text. Data on airway improvement, decannulation rate, voice outcome, swallowing function, and reported complications were extracted. In addition to the methodological characteristics of the studies, the authors reported the sample size, age, and gender, as well as the advantages and disadvantages.

Risk of Bias

Eligible articles were assessed for their quality by two independent reviewers, and conflicts were resolved through discussion at the end of the process. We employed appropriate tools to evaluate the methodical quality of each of the three included designs. Case reports were evaluated against the criteria provided by Murad et al. [31]. Case series were assessed using the NIH Quality Assessment Tool for Case Series Studies [32]. Finally, retrospective cohort studies were tested using the NIH Quality Assessment Tool for Observational Cohort and Cross-Sectional Studies [33].

Statistical Analysis

This analysis was performed via OpenMeta v5.26.14 software. The meta-analysis was performed to compute the overall decannulation rate for SGS, PGS, and BVFI combined with the respective 95% CI. We considered the p-value less than 0.05 to be statistically significant. We calculated the heterogeneity of the included studies using the I² statistic, where values of 25%, 50%, and 75% mark the cutoff for low, moderate, and high heterogeneity, respectively. We employed the random effects model to account for the wide spectrum of variations in the complexity and severity of pathology. The results were depicted on a forest plot, and heterogeneity was assessed using several methods, including the Tau2, the Q Cochrane test (Q-test), and the I² statistic.

Results

**Figure 1:**
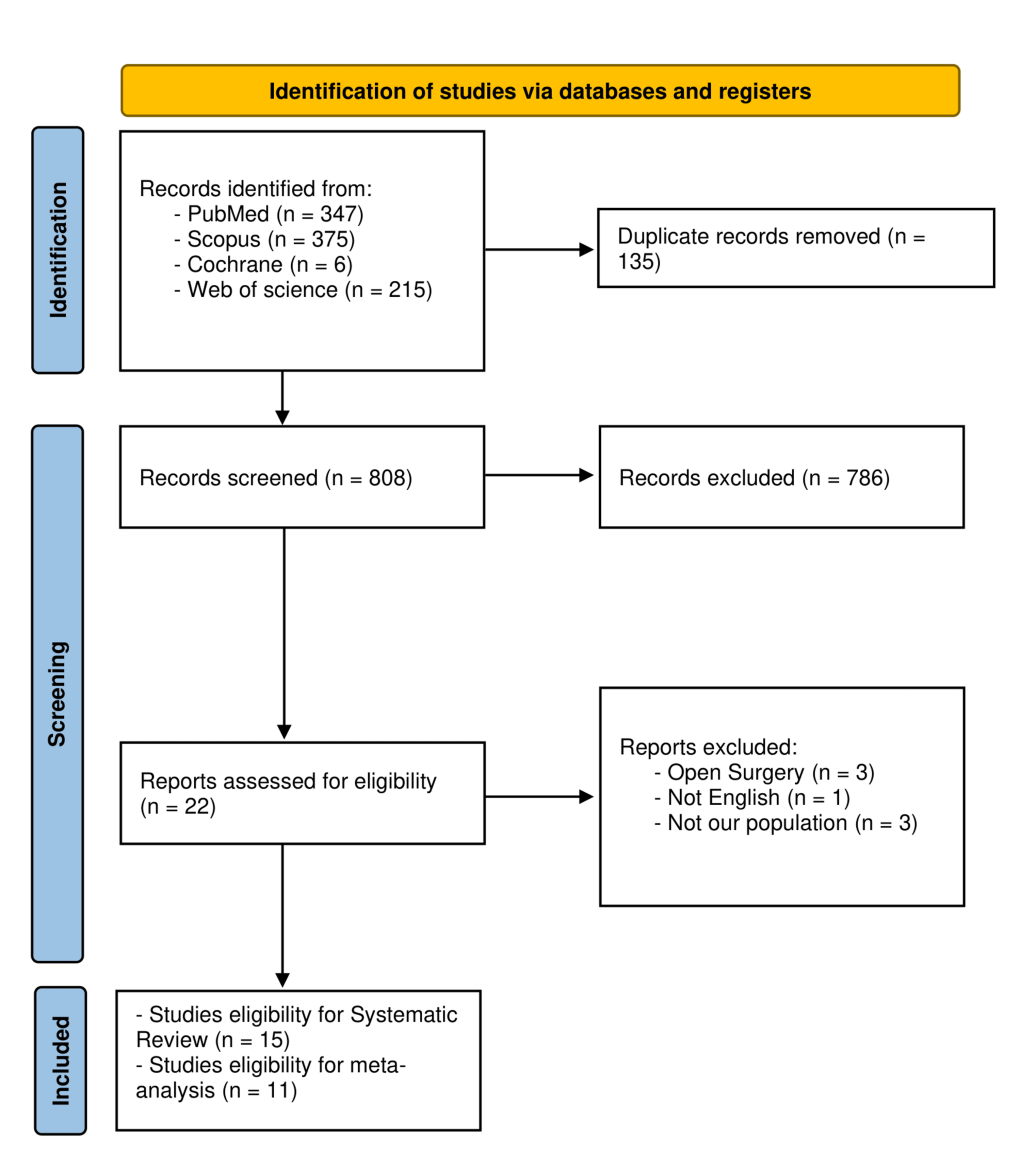
PRISMA flowchart PRISMA, Preferred Reporting Items for Systematic reviews and Meta-Analyses

Article selection went through three steps. First, we retrieved 943 records from searching four databases; 135 records were discarded as being duplicates. Secondly, we screened the titles and abstracts of the resulting 808 records; this process resulted in the exclusion of 786 records. Lastly, we scrutinized 22 articles in full text; only 15 articles were eligible for inclusion in the review. Subsequently, 11 studies made it into the analysis [20,21,34-46]. The process of article selection is presented in Figure 1.

Characteristics of the Included Studies

Eligible articles included four retrospective cohort studies, six case series, and five case reports. A total of 144 patients underwent endoscopic posterior cricoid split with posterior grafting, with an average age ranging from 11 months to 14.2 years. The underlying pathology was BVFI in 42 cases, PGS in 49 cases, and SGS in 22 cases, in addition to 31 cases with more than one pathology. Most studies were undertaken in the USA (11), followed by the UK (2), Brazil (1), and Saudi Arabia (1). The outcomes reported were decannulation rate (11/15), complications (7/15), subsequent airway procedures (4/15), resolution of symptoms (3/15), hospital stay (4/15), laryngeal function (1/15), and pulmonary function test parameter (1/15) (Table 1).

**Table 1:** Summary and baseline characteristics of the included studies BVFI: bilateral vocal fold insufficiency; BVFP: bilateral vocal fold paralysis; CAJF: cricoarytenoid joint fixation; NR: not reported; PGS: posterior glottic stenosis; SGS: subglottic stenosis; VCI: vocal cord insufficiency

Author	Study design	Country	Number of patients	Diagnosis, N	Age, mean in years	Male, N (%)	Prematurity, N (%)	Hospital stay, means in days	Outcomes	Conclusion
Aldriweesh and AlAmmar (2022) [34]	Case series	Saudi Arabia	5	PGS, 3; SGS (grade 2), 1; SGS (grade 2) + PGS, 1	14.2	4 (80%)	NR	14.6	(1) Pulmonary function test parameters	“The present surgical technique modification is advantageous in overcoming the difficult step of costal cartilage graft placement. It is potentially applicable to a broader age group. With the aid of balloon expansion force, the graft locks into the divided cricoid lamina. Potential risks of balloon dilation include airway laceration, pneumomediastinum, pneumothorax, and, rarely, entrapment of the inflated balloon in the airway. All patients described in this series underwent single-stage repair. Tracheostomy and its complications were avoided.”
Bajaj et al. (2011) [35]	Case series	United Kingdom	2	PGS, 2	11.5	NR	NR	NR	(1) Successful rate; (2) Complications; (3) Requiring tracheostomy	“Single-stage laryngotracheal reconstruction with endoscopic placement of the posterior graft in cases with isolated PGS is a good alternative to open surgical techniques, although it is technically a challenging procedure.”
Dahl et al. (2017) [36]	Retrospective cohort	USA	32	PGS, 12; SGS, 13; BVFI, 7	5.15	NR	22 (68.75%)	3	(1) Decannulation rate; (2) Length of hospitalization; (3) Subsequent airway procedures	“This represents the largest series of patients who have undergone EPCS/RG and demonstrates that the majority of patients can be decannulated after this procedure. Patients with PGS had the highest operation-specific decannulation rates.”
Evans et al. (2016) [37]	Case report	United Kingdom	6	PGS + VCI, 5; PGS + SGS, 1	10.33	3 (50%)	6 (100%)	9.8	(1) Resolution of the symptoms; (2) Complications	“Our experience shows that endoscopic posterior laryngotracheal reconstruction with costal cartilage grafting is an effective means of widening stenoses at the level of the posterior glottis. It is a valuable alternative to open reconstruction and avoids the potential morbidities associated with laryngofissure. It is a non-destructive procedure compared to cordotomy or arytenoidecotmy with encouraging voice outcomes. It can also be performed in the absence or presence of a preexisting tracheostomy.”
Gerber et al. (2013) [38]	Case series	USA	28	PGS, 3; SGS, 8; BVFI, 9; PGS+ SGS, 8	4.55	16 (57.14%)	NR	NR	(1) Decannulation rate; (2) Complications; (3) Subsequent airway procedures	“This descriptive series shows a consistent outcome in more than double the number of cases previously reported in the previously published series. We believe that EPCSCG is an important option to have in the management of pediatric glottis/SGS and BVFI.”
Inglis et al. (2003) [20]	Case series	USA	10	PGS, 4; PGS + SGS, 6	2.49	NR	NR	6.29	(1) Decannulation rate; (2) Laryngeal function; (3) Hospital stay	“EPCS/RG appears to be safe and effective in the management of PGS in selected pediatric patients. This minimally invasive procedure has advantages over traditional open approaches and destructive endoscopic techniques (cordotomy and arytenoidectomy). The role of EPCS/RG alone in the face of severe grades of SGS appears to be limited.”
Jang and Grunstein (2021) [21]	Case series	USA	3	PGS, 2; PGS + SGS, 1	10.33	2 (66.67%)	1 (33.33%)	9.5	(1) Resolution of the symptom; (2) Subsequent airway procedures	“We conclude that for patients with posterior laryngeal stenosis but without tracheostomy, EPCS/RG with endotracheal tube stenting might be a safe option. Further studies with larger patient samples are needed.”
Modi (2012) [39]	Case report	USA	2	BVFI, 2	1.75	NR	2 (100%)	NR	(1) Decannulation rate	“The management of BVFI in pediatric patients is a multifaceted challenge that necessitates a tailored approach to each case. The EPCG has proven to be an effective intervention, as demonstrated in the presented cases. In Case 1, a 1.5-year-old child with CAJF, PGS, and grade 2 SGS underwent successful EPCG, highlighting the procedure’s ability to address multiple aspects of airway obstruction without compromising laryngeal function. Similarly, Case 2, involving a two-year-old child with BVFP, showed favorable outcomes with the EPCG, leading to decannulation within two months postoperatively.:
Bressan Pazinatto et al. (2021) [40]	Case report	Brazil	1	BVFI, 1	3	1 (100%)	NR	NR	(1) Decannulation rate	“As a less invasive surgical approach, the endoscopic posterior cricoid split with cartilage grafting procedure is a good option for BVFI. Sutures are not necessary when the graft is well-positioned and carving lateral flanges is possible. The risk of graft dislodgement and airway obstruction is mitigated by short-term stenting with endotracheal intubation and/or a tracheostomy.”
Provenzano et al. (2011) [41]	Retrospective cohort	USA	12	PGS, 3; PGS + SGS, 9	7.4	6 (50%)	NR	3.5	(1) Decannulation rate; (2) Complications	“Endoscopic posterior cricoid grafting is a valuable surgical option for patients with PGS. The procedure is associated with low morbidity and permits decannulation in the majority of patients.”
Redmann et al. (2023) [42]	Retrospective cohort	USA	21	PGS, 13; BVFI, 8	5.9	13 (61.9%)	5 (23.8%)	NR	(1) Decannulation rate	“Our results for EPCCG are consistent with those reported in the literature and suggest that EPCCG can safely be performed in revision cases in select patients with PGS or BVTI. A larger multicenter study would be helpful to better define which patients and indications are best suited for this procedure.”
Richard et al. (2024) [46]	Retrospective cohort	USA	21	BVFI, 21	27.7	8 (38.1%)	NR	9	(1) Decannulation rate; (2) Complications; (3) Resolution of the symptoms	“EnPCCG was more successful at achieving decannulation in children. Adults required additional interventions. A double-staged operation with prolonged stenting is recommended for adult patients. A majority of patients were decannulated at last follow-up.”
Shah et al. (2018) [43]	Case report	USA	2	PGS, 1; BVFP, 1	2.45	2 (100%)	NR	5	(1) Decannulation rate; (2) Complications	“The proposed endoscopic suturing technique for graft securement could reduce the risk of graft extrusion in EPCSCG, allowing for early or same-day extubation and avoiding the need for prolonged intubation or tracheostomy in these patients. Given the decreased duration of postoperative intubation, it would also likely result in comparably shorter lengths of ICU stay and overall hospitalization. To our knowledge, this is the only report of a sutured graft in endoscopic posterior cricoid expansion. Further studies will be needed to demonstrate outcomes associated with the endoscopic suturing technique described.”
Thakkar and Gerber (2008) [44]	Case report	USA	2	BVFP, 2	2-year newborn baby	NR	NR	NR	(1) Decannulation rate; (2) Complications	“The management of acute BVP with upper airway obstructive symptoms usually requires tracheotomy. We present an extension of a previously described technique to endoscopically separate the arytenoids using an endoscopically placed costal cartilage graft as an alternate approach in the pediatric population with minimal risk to phonation and aspiration.”

Risk of Bias

The quality of case reports was addressed using the criteria provided by Murad et al [31]. All five studies had scores between 5 and 6, which indicates fair quality. Case series studies were evaluated using the NIH tool, and the scores ranged between 5 and 7.5. The scores were categorized as follows: Good (7.5 to 9 points), Fair (5 to 7 points) or Poor (0 to 4.5 points). As for cohort studies, the scores fell between 8 and 9, indicating fair quality (Table 2, Table 3, Table 4).

**Table 2:** Murad et al. (2018)’s assessment tool for case report studies CD: cannot determine; NA: not applicable; NR: not reported

Author	(1) Does the patient represent the whole experience of the investigator (center), or is the selection method unclear to the extent that other patients with similar presentation may not have been reported?	(2) Was the exposure adequately ascertained?	(3) Was the outcome adequately ascertained?	(4) Were other alternative causes that may explain the observation ruled out?	(5) Was there a challenge/rechallenge phenomenon?	(6) Was there a dose-response effect?	(7) Was follow-up long enough for outcomes to occur?	(8) Is the case described with sufficient details to allow other investigators to replicate the research or to allow practitioners make inferences related to their own practice?	Total scores (Yes = 1; No = 0.5; NR and NA and CD = 0)	Quality rating (Good: 6.5-8 points; Fair: 5-6.5 points; Poor: 4.5-0 points)
	Yes/No/NR or CD or NA	Yes/No/NR or CD or NA	Yes/No/NR or CD or NA	Yes/No/NR or CD or NA	Yes/No/NR or CD or NA	Yes/No/NR or CD or NA	Yes/No/NR or CD or NA	Yes/No/NR or CD or NA		
Evans et al. (2016) [37]	Yes	Yes	Yes	NA	Yes	NA	Yes	Yes	6	Fair
Modi (2012) [39]	Yes	Yes	Yes	NA	Yes	NA	NR	Yes	5	Fair
Bressan Pazinatto et al. (2021) [40]	Yes	Yes	Yes	NA	Yes	NA	NR	Yes	5	Fair
Shah et al. (2018) [43]	Yes	Yes	Yes	NA	Yes	NA	Yes	CD	5	Fair
Thakkar et al. (2008) [44]	Yes	Yes	Yes	NA	Yes	NA	No	Yes	5.5	Fair

**Table 3:** NIH quality assessment tool for observational case series studies CD: cannot determine; NA: not applicable; NR: not reported

Author	(1) Was the study question or objective clearly stated?	(2) Was the study population clearly and fully described, including a case definition?	(3) Were the cases consecutive?	(4) Were the subjects comparable?	(5) Was the intervention clearly described?	(6) Were the outcome measures clearly defined, valid, reliable, and implemented consistently across all study participants?	(7) Was the length of follow-up adequate?	(8) Were the statistical methods well-described?	(9) Were the results well-described?	Total scores (Yes = 1; No = 0.5; NR and NA and CD = 0)	Quality rating (Good: 7.5-9 points; Fair: 5-7 points; Poor: 4.5-0 points)
	Yes/No/NR or CD or NA	Yes/No/NR or CD or NA	Yes/No/NR or CD or NA	Yes/No/NR or CD or NA	Yes/No/NR or CD or NA	Yes/No/NR or CD or NA	Yes/No/NR or CD or NA	Yes/No/NR or CD or NA	Yes/No/NR or CD or NA		
Aldriweesh and AlAmmar (2022) [34]	Yes	Yes	CD	CD	Yes	Yes	Yes	No	Yes	6.5	Fair
Bajaj et al. (2011) [35]	Yes	Yes	CD	Yes	Yes	Yes	Yes	No	Yes	7.5	Good
Gerber et al. (2013) [38]	Yes	Yes	No	No	Yes	Yes	Yes	No	Yes	7.5	Good
Inglis et al. (2003) [20]	Yes	Yes	CD	No	Yes	Yes	Yes	No	Yes	7	Fair
Jang and Grustein (2021) [21]	Yes	Yes	CD	CD	Yes	Yes	NR	No	No	5	Fair
Valika et al. (2024) [45]	Yes	Yes	CD	Yes	Yes	Yes	Yes	No	Yes	7.5	Good

**Table 4:** quality assessment tool for observational cohort CD: cannot determine; NA: not applicable; NR: not reported

Author	(1) Was the research question or objective in this paper clearly stated?	(2) Were eligibility/selection criteria for the study population prespecified and clearly described?	(3) Were the participants in the study representative of those who would be eligible for the test/service/intervention in the general or clinical population of interest?	(4) Were all eligible participants who met the prespecified entry criteria enrolled?	(5) Was the sample size sufficiently large to provide confidence in the findings?	(6) For the analyses in this paper, were the exposure(s) of interest measured prior to the outcome(s) being measured?	(7) Was the time frame sufficient so that one could reasonably expect to see an association between exposure and outcome if it existed?	(8) For exposures that can vary in amount or level, did the study examine different levels of the exposure as related to the outcome (e.g., categories of exposure, or exposure measured as continuous variable)?	(9) Were the exposure measures (independent variables) clearly defined, valid, reliable, and implemented consistently across all study participants?	(10) Was the exposure(s) assessed more than once over time?	(11) Were the outcome measures prespecified, clearly defined, valid, reliable, and assessed consistently across all study participants?	(12) Were the people assessing the outcomes blinded to the participants' exposures/interventions?	(13) Was the loss to follow-up after baseline 20% or less? Were those lost to follow-up accounted for in the analysis?	(14) Were key potential confounding variables measured and adjusted statistically for their impact on the relationship between exposure(s) and outcome(s)?	Total scores	Quality rating (Good: 11-14 points; Fair: 7.5-10.5 points; Poor: 0-7 points; Yes = 1; No = 0.5; NR and NA and CD = 0)
	Yes/No/NR or CD or NA	Yes/No/NR or CD or NA	Yes/No/NR or CD or NA	Yes/No/NR or CD or NA	Yes/No/NR or CD or NA	Yes/No/NR or CD or NA	Yes/No/NR or CD or NA	Yes/No/NR or CD or NA	Yes/No/NR or CD or NA	Yes/No/NR or CD or NA	Yes/No/NR or CD or NA	Yes/No/NR or CD or NA	Yes/No/NR or CD or NA	Yes/No/NR or CD or NA		
Dahl et al. (2017) [36]	Yes	Yes	Yes	Yes	CD	Yes	Yes	NA	Yes	NA	Yes	NR	Yes	NR	9	Fair
Provenzano et al. (2011) [41]	Yes	Yes	Yes	Yes	CD	Yes	Yes	NA	Yes	NA	Yes	NR	Yes	NR	9	Fair
Redmann et al. (2023) [42]	Yes	Yes	Yes	Yes	No	Yes	Yes	NA	Yes	NA	Yes	NR	Yes	NR	9.5	Fair
Richard et al. (2024) [46]	Yes	Yes	Yes	Yes	No	Yes	Yes	NA	Yes	NA	Yes	NR				

Results of syntheses

Decannulation

**Figure 2:**
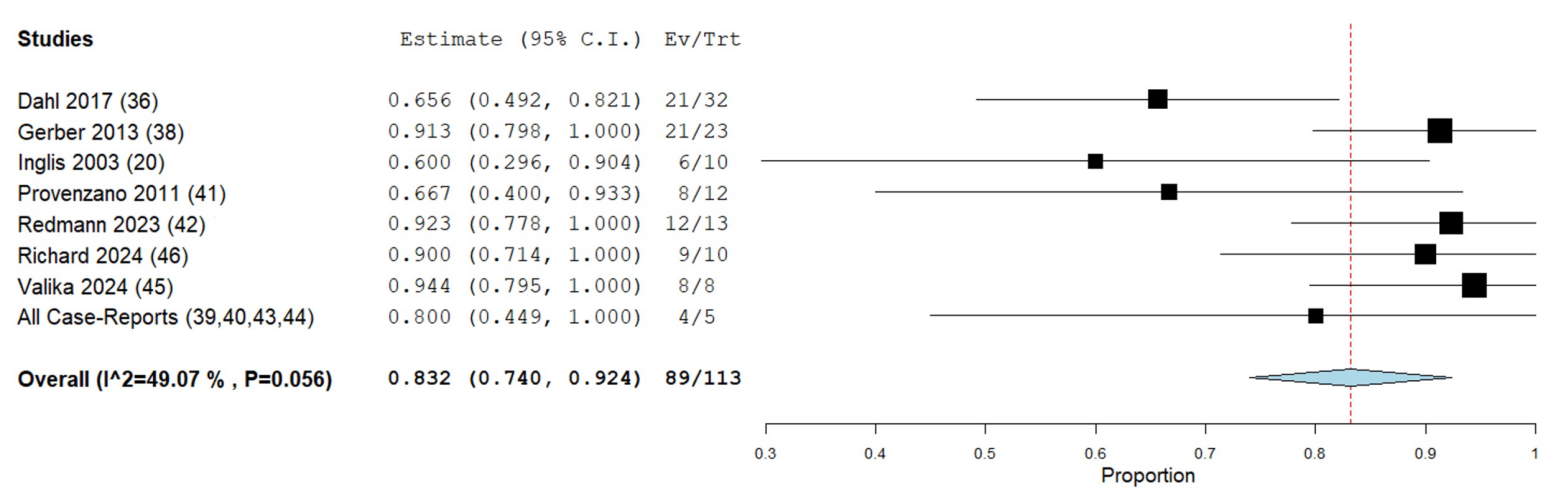
Forest plot of decannulation rate

The analysis indicates that the EPCCG has a statistically significant and effective decannulation rate, with an estimated proportion of approximately 83.2% (95% CI: 74.0-92.4%). The observed heterogeneity (I²: 49.07%; p-value: 0.056) among the studies is low and statistically significant, suggesting a heterogeneity effect across the included studies (Figure 2).

Complications

Across the studies, several complications were reported postoperatively. Evans et al. noted complications, including opiate withdrawal, pneumothorax, lower respiratory tract infection, recurrent stridor, and glomerulonephritis [37]. Gerber et al. documented four patients who needed early manipulation for symptom management or decannulation; one required graft replacement due to displacement; three underwent dilations, with one requiring endoscopic stenting; and two needed additional surgeries to address residual issues [38]. Shah et al. reported no postoperative complications, and the patient was discharged on the sixth postoperative day [43]. Thakkar et al. described delayed graft placement by two days due to intraoperative complications with sevoflurane and noted no significant improvement in one patient after 20 days of observation following tracheal resection [44]. Richard et al. observed one emergency department visit within 30 days post-operation [46].

Need for Further Airway Procedures

In the studies reviewed, the necessity for airway procedures varied among patients, as reported by different authors. Bajaj et al. and Evans et al. found that none of their patients required tracheostomy during or after surgery [35,37]. Gerber et al. documented specific cases where additional procedures were necessary, such as cordotomy or managing exercise limitations post-surgery, which was managed by unilateral vocal fold suture lateralization [38]. Provenzano et al. noted one instance where a second tracheostomy was needed, along with previous reconstructive procedures [41]. Shah et al. observed successful extubation in some patients without tracheostomy and effective healing of grafts in others [43]. Dahl et al. found that 38.5% of SGS patients had subsequent open surgery, 15.4% needed a second EPCCG procedure, and 7.7% required both. PGS patients required open surgery in 8.3% of cases and a second EPCCG procedure in 8.3% [36].

Resolution of Symptoms

Multiple outcomes were reported in terms of symptom resolution post-surgery. Evans et al. found that five patients experienced immediate symptom relief following the procedure. However, one child experienced worsened stridor due to scarring and restenosis post-surgery. This issue was resolved after undergoing two additional endoscopic procedures [37]. Richard et al. noted that, at the last follow-up, none of the patients were aphonic, but six out of 10 patients expressed concerns with voicing. Postoperative videofluoroscopic swallow studies (VFSS) were conducted for five patients due to swallowing concerns, with one patient showing intermittent aspiration on VFSS that later resolved and another experiencing pain with swallowing. Still, they had a normal VFSS [46]. Gerber et al. mentioned that all three patients with isolated PGS or cricoarytenoid joint fixation were decannulated with reasonable symptom control [38]. Shah et al. reported complete resolution of stridor and improved voice with the patient tolerating a regular diet without issues at the one-month follow-up [43]. Jang et al. described varied outcomes across three cases: in Case 1, symptoms resolved with a patent airway at one-year post-op; in Case 2, breathing significantly improved at one-month post-op, with resolved stridor and improved exercise tolerance at one-year post-op; and in Case 3, improved dysphagia was noted at five months post-op, and stridor had resolved with improved exercise tolerance at one-year post-op [21].

Length of Hospital Stay

Included studies reported varied lengths of hospital stay post-surgery. Evans et al. reported a mean total inpatient stay of 9.8 days [37]. Provenzano et al. provided two sets of data for the length of stay: including new tracheostomies, the length of ICU stay was 2.1 days, and hospitalization was 3.6 days; excluding new tracheostomies, the ICU stay was 1.2 days, and hospitalization was 2.6 days [41]. Dahl et al. reported a median length of hospitalization after EPCS/RG as three days [36]. Richard et al. noted that patients remained hospitalized for an average of 11.2 days [46].

Discussion

Endoscopic techniques are changing the management landscape of airway compromise. EPCCG provides hope for permanent decannulation of patients with SGS, PGS, and BVFI. Here, we provide a synopsis of our findings and highlight the milestones of EPCCG development.

We observed a statistically significant decannulation rate of 83.2% associated with EPCCG. Some included articles reported decannulation rates higher than 90%, and others reported rates below 70%. Both Redmann et al. and Gerber et al., who reported rates higher than 90%, had most patients with isolated pathologies [38,42]. Provenzano et al. reported a rate of 66.7%, including 9/12 patients with combined SGS+PGS [41]. Dahl et al. reported a rate of 65.6% divided into 53.8% for SGS, 100% for PGS, and 28.6% for BVFI. The authors attributed the higher success with PGS patients to the absence of preoperative need for tracheostomy, suggesting less severe pathology. Additionally, the authors claim that if they excluded patients with comorbidities and those lost to follow-up, the rate would rise to 80% [36]. Inglis et al. had a 60% decannulation rate, which may be attributed to the fact that most of their patients either had advanced stenosis or were tracheostomy-dependent preoperatively [20].

Complications were not common after EPCCG, with only six patients having procedure-related complications out of 70 patients for whom complications were reported. Gerber et al. detailed early interventions for symptom management in four patients, including graft replacement, dilations, and additional surgeries [38]. Thakkar et al. observed no improvement in one patient after 20 days post-tracheal resection observation [44]. Richard et al. observed one emergency department visit within 30 days post-operation [46]. Overall, no mortality was reported, and most patients could be managed successfully; these findings assert the procedure’s safety. Moreover, hospital stays ranged between 3 and 9.8 days, with an average of 6.1 days in three studies reporting the outcome [36,37,41].

Few patients required additional airway procedures after EPCCG. Sixteen out of 88 patients in studies reporting the outcome required some sort of airway procedure to achieve decannulation or to address complications. Gerber et al. detailed that one patient underwent cordotomy, leading to subsequent decannulation, while another developed exercise limitations four years post-EPCCG and later managed with unilateral vocal fold suture lateralization [38]. Provenzano et al. noted one patient required a second tracheostomy due to scar tissue post-decannulation [41]. Dahl et al. highlighted that in the SGS group, 38.5% needed open airway surgery, 15.4% required a second EPCS/RG, and 7.7% needed both. In the PGS group, 8.3% required open surgery, and 8.3% needed a second EPCS/RG [36].

EPCCG underwent multiple modifications since its inception by Inglis et al. in 2003. Inglis et al. used a CO2 laser to make a vertical midline incision in the posterior cricoid plate regarding cricoid splitting [20]. Graft harvesting was performed by Inglis et al. from the eighth rib, whereas other authors preferred the fifth rib. Provenzano et al. mentioned using autologous rib harvest as the preferred method, with the option of cadaveric cartilage [41].

This study is the first attempt to assemble evidence regarding the different outcomes of EPCCG. We were able to draw some broad conclusions about the success and safety of the procedure. Nonetheless, the number of subjects in each outcome and the study remains small. The fact that many patients had more than one pathology and the presence of comorbidities limits the generalizability of these results to the broader population with laryngotracheal stenosis. More extensive studies with standardized protocols are required to address the shortcomings of available evidence.

## Conclusions

EPCCG shows considerable potential as an innovative approach for managing less severe cases of PGS, SGS, and BVFI. The technique is associated with a strong safety profile, reduced hospital stays, and effective symptom relief, making it a valuable addition to the arsenal of minimally invasive airway management options. These benefits position EPCCG as an appealing choice for specific patient groups.

However, the application of EPCCG in cases involving advanced pathology or patients with significant comorbidities remains uncertain. To fully realize its potential in complex scenarios, further research is needed to refine its use and evaluate long-term outcomes. Addressing these areas will help determine the broader utility of EPCCG and enhance its role in comprehensive airway care.
